# Evaluation of Reinforced Concrete Structures with Magnetic Method and ACO (Amplitude-Correlation-Offset) Decomposition

**DOI:** 10.3390/ma16165589

**Published:** 2023-08-12

**Authors:** Paweł Karol Frankowski, Tomasz Chady

**Affiliations:** Faculty of Electrical Engineering, West Pomeranian University of Technology in Szczecin, ul. Sikorskigo 37, 70-313 Szczecin, Poland

**Keywords:** reinforcement bars detection, rebars, concrete inspection, reinforced concrete, ACO decomposition, pattern recognition, attributes extraction, nondestructive testing NDT, nondestructive evaluation NDE, anisotropic magneto-resistance (AMR) sensor, signal processing

## Abstract

The magnetic method is one of the very few nondestructive testing (NDT) techniques that provide the possibility to conduct area tests of reinforced concrete (RC) structures in a fast, cheap, and straightforward way. This paper aims to present a new approach to the simultaneous identification of rebars’ diameter, alloy class, and thickness of the concrete cover tested with this method. Since rebars from different manufacturers may have different electromagnetic properties (standardization only for mechanical properties), preparing an effective and universal database is impossible. In this work, ACO decomposition is proposed, a new attributes extraction method designed to identify object parameters, even if it is impossible to collect a suitable training database (by pattern recognition and analysis of the deviation). Conducted tests prove that the ACO method enables accurate reflection of the waveform shape and limitation of attributes number to three or fewer (avoiding the curse of dimensionality). These properties, combined with the ability to analyze spatial components of magnetic induction (which only magnetic sensors provide), make the complex task of identification of three parameters more straightforward and the separation between the results received for different classes larger. This article presents the measurement results and the whole identification process.

## 1. Introduction

Reinforced concrete has been a versatile, dominant construction material worldwide for over a century. The lifetime of structures constructed using this material is difficult to estimate due to various factors and external conditions influencing the lifetime of a specific construction. However, buildings of this kind were usually designed for 50–100 years of operating time. Many structures built at the beginning of the twentieth century are still in service, and their remaining life is ending. Therefore, periodic inspections are necessary and usually required by a building code. Moreover, even in the case of new constructions, acceptance tests are conducted to determine if the requirements of a specification or contract are met. For these reasons, developing new NDT (nondestructive testing) methods designed to inspect RC (reinforced concrete) structures is becoming increasingly important [1,2].

As described in [3,4], corrosion is the biggest problem in exploiting RC structures. Most papers about evaluating RC structures published in the last few years are strictly related to this topic. Nonetheless, even if most of the NDT methods are designed mainly to evaluate concrete, the methods can also be used to estimate concrete cover thickness, detect reinforced bars (rebars) diameter, or steel class (the class determines the hardness/ductility of the steel). The description and comparison of NDT methods used in civil engineering are provided in [3].

### 1.1. Nondestructive Testing (NDT) of Reinforced Concrete (RC) Structures

**Figure 1:**
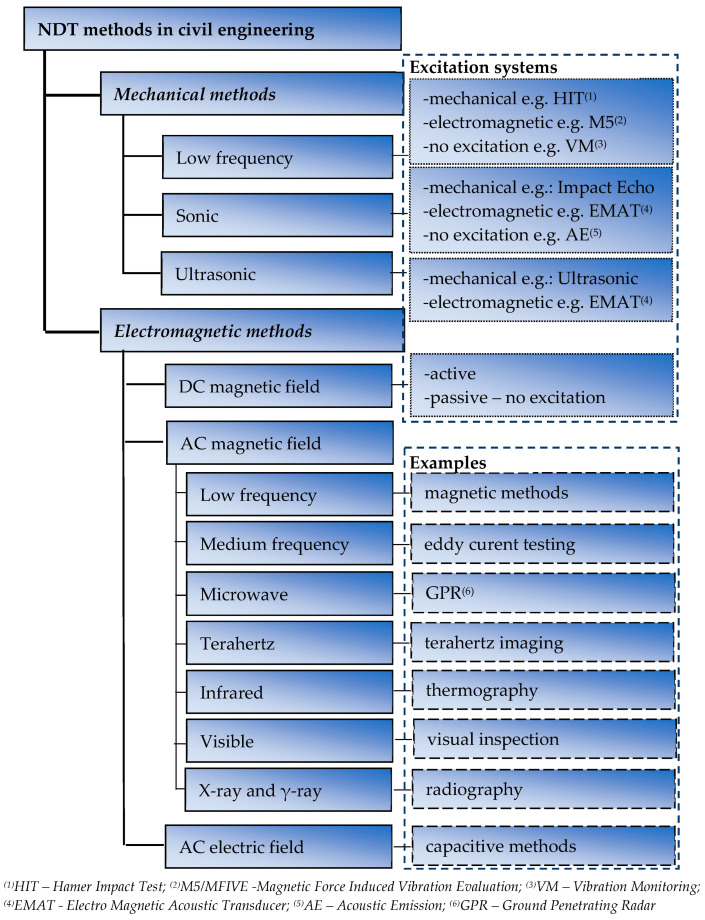
Mechanical and electromagnetic NDT methods used in civil engineering.

Two groups of methods can be effectively and directly used for the reinforcement assessment [3]. The first, more popular group is based on mechanical waves. In the other group, electromagnetic waves are utilized. In the case of mechanical-wave methods, both electromagnetic and mechanical systems can be used to generate the waves. In magnetic or electromagnetic methods, the waves are generated using electromagnetic transducers. Both the magnetic and the mechanical methods can be active or passive. A short overview of NDT methods used in civil engineering is shown in Figure 1.

One of the crucial factors in electromagnetic and mechanical methods is the excitation frequency. Depending on the frequency, the same technique may deliver a high spatial resolution and a short range or a long effective and low-resolution range. Usually, the size of minor defects that can be detected is comparable to the wavelength of the excitation [3].

In the case of the evaluation of reinforced concrete structures, the significant disadvantage of mechanical methods is that the results depend on many factors, and various phenomena may strongly affect the propagation of mechanical waves in complex structures. Moreover, a relatively narrow frequency band (due to the limitation of the maximum frequency) can be applied. Considering the waves’ frequency, three categories of mechanical methods can be distinguished [3]:

- Methods based on the ultrasonic frequencies (typically ≥ 20 kHz) may utilize mechanical and electromagnetic excitation systems. Mechanical excitation with piezo-ceramic transducers is much more popular. This group of methods is universal and can be used to detect rebar position [5,6], rebar parameters, or even rebar corrosion [7]. Applying a pulse compression technique and post-processing (e.g., Total Focusing Method—TFM) allows for obtaining reliable maps of reinforcement even for concrete cover thickness above 0.5 m. The methods are used primarily to determine the quality of the concrete [3,8,9,10,11,12]. External excitation can be unnecessary in some methods, such as Acoustic Emission (AE) testing. The AE is the passive technique that monitors the release of sonic waves generated when a material deforms under stress. The AE is commonly used in industrial applications to detect cracks, monitor weld quality, test structural integrity, detect leaks, and as a system for structural health monitoring (SHM). Systems of this kind can only qualitatively gauge structure damage and are noise-sensitive [3,13].
- Methods based on sound frequencies (typically from 20 Hz to 20 kHz) and low frequencies (typically < 20 Hz). These two groups have been combined because, in terms of frequency, the methods often cannot be classified into one of the groups and overlap with both. The methods can be categorized similarly to the previous group. An excellent example of the active sonic-frequency mechanical method is Impact-Echo (IE). IE is an NDT method for testing concrete and masonry structures. The method uses impact-generated stress waves that propagate through the structure and are reflected by the internal borders, flaws, and external surfaces. Transducers with electromagnetic excitation can usually work both sonic and ultrasonic frequencies. The lower frequency methods usually used in civil engineering are HIT (Hammer Impact Test) and shaker (vibration tester). The excitation is usually mechanical, but many methods with electromagnetic excitation are increasingly encountered. In these cases, the identification is mainly based on modal analysis (vibration testing of an object whereby the natural frequencies and damping ratios are determined). Good examples of mechanical methods with electromagnetic excitation are M5 [3,14,15,16] and EPAT [17]. Passive methods in the sonic-frequency range are usually used to study sounds generated during the regular operation of the device. A typical example is the noise test on transformers. In lower frequencies, the seismic vibration monitoring (VM) is working.

Electromagnetic and magnetic testing methods have many advantages. The most significant is that the concrete cover above the rebar is practically transparent for waves of this kind (in contrast to mechanical waves, where the damping is high) and usually does not significantly affect measurement results. Some of the electromagnetic methods enable the impact of rebars directly. Therefore, these methods are recommended for evaluating rebars in RC structures. The range of the excitation frequency can be used to distinguish different electromagnetic methods of NDT:

- Methods based on X and γ radiation frequencies (frequencies of the order of 10^16^ Hz to 10^24^ Hz). Radiography is a very effective method, but it has some limitations and is rarely used to evaluate RC structures. The method may create risks to human health. There is also a problem with accessing both sides of the tested element. The source and detector must usually be placed on both sides of the tested object (the exception is backscattering), which is, in many cases, challenging to implement [3].
- Methods based on visible light frequencies (frequencies of the order of 10^14^ Hz to 10^15^ Hz). In this frequency range, strictly electromagnetic methods are not often used. However, these frequencies are used in visual inspection (VI). VI is a preliminary technique commonly used before other, more accurate NDT methods. VI is limited to evaluating the external conditions of the structure [3,18].
- Methods based on infrared radiation frequencies (frequencies of the order of 10^11^ Hz to 10^14^ Hz). Similarly to the previous group, in this frequency range, electromagnetic methods are not often used. Infrared thermography (IR) is more popular. IR is an important method that can potentially be used to test reinforced concrete. This method is mainly used to test the concrete; however, it is one of the very few that can also be implemented as an area-testing method for the initial detection of rebars in large-sized structures [3] and sometimes to detect corrosion. The method’s effectiveness strongly depends on the thickness of the concrete cover. Essential for effectiveness is the method of heating. The method can be helpful when the cover thickness is lower than 5 cm. The method is not commonly used in practice [3,19,20,21,22,23,24].
- Methods based on terahertz frequencies (frequencies of the order of 10^11^ Hz to 10^13^ Hz). The terahertz technique is rarely used due to the very high equipment price and limited penetration of concrete (caused by water content in concrete which damps waves of that frequency) [3].
- Methods based on microwave frequencies (frequencies of the order of 10^9^ Hz to 10^11^ Hz). The most crucial method in this group is ground-penetrating radar (GPR). GPR is an up-and-coming method. Rebars can be detected from several centimeters up to ten or more meters, while other electromagnetic methods usually have a maximum detection range below 20 cm. In some cases, GPR can be utilized to estimate the diameter of rebars, detect breaks and defects, or even for corrosion detection (debonding). The method may also be applied to mapping multilayer reinforced meshes. Many factors (such as voids or variable internal moisture conditions) may affect the results of this technique. The other disadvantages of GPR are the cost of the device, difficulties with results interpretation, and limited resolution [3,25,26,27,28,29,30].
- Methods based on low and medium frequencies (frequencies of the order of 10^2^ Hz to 10^9^ Hz). The eddy current (EC) method is one of the most important electromagnetic methods in civil engineering. The method can be applied to detect rebars’ presence and determine: position, diameter, and rebar alloy (due to different electrical properties) [31,32,33,34,35]. In some cases, the method can detect changes caused by corrosion [36,37,38,39]. Both single and multifrequency excitation can be applied in various types of methods [39]. Because the EC method is sensitive to external interferences, it is essential to use appropriate algorithms, especially when many parameters must be identified simultaneously [40,41,42,43]. The effective range of the method is from 0 to 60 mm [31,32,33,34,35,36,37,38,39,39,40,41,42,43,44]. Similarly, the capacitive method works in this frequency range like in the EC method case. Sensors of this type usually can detect smaller inhomogeneities, but their effective range is also smaller than that of the EC method [45,46,47].
- Methods based on DC magnetic field—a large group of various methods. Generally, the methods can be divided into two groups: Continuous Magnetization Techniques (CMT), where an excitation device is utilized, and Residual Magnetization Techniques (RMT), which is passive. The main representative of CMT is the Magnetic Flux Leakage method (MFL). The method can be applied in civil engineering. However, the central area of use is in producing ferromagnetic parts and components. MFL can be used to localize rebars in the structure. Moreover, the propagation of magnetic flux can be obstructed by discontinuities in the material, such as breaks or cracks. Therefore, MFL may also be utilized to detect defects in rebars. In some cases, the MFL method can be used even to find out the material loss caused by corrosion. Other active magnetic methods, such as Barkhausen emission, magneto-acoustic emission, stress-induced magnetic anisotropy, or magnetic powder method, are usually not applied to evaluate RC structures. The CMT methods are more sensitive than RMT because the leakage field is higher when the magnetic flux is excited. However, the method poses some disadvantages, such as equipment deployment and power consumption. Residual magnetization methods are more economical and straightforward. One of the RMTs is the Magnetic Memory Method (MMM). The method can detect abnormal conditions arising from changes in crystalline structures of rebar material resulting from stress concentration, corrosion, or cracks [3,48,49,50,51,52,53,54,55]. The magneto-resistance (MR) and Hall effect sensors are most commonly used in the magnetic test. The MR sensors are usually susceptible and well-fitted for measuring small magnetic fields [56]. The Hall effect sensors are less sensitive and more appropriate for measuring relatively high magnetic fields. The magnetic method is one of the very few that can be implemented as an area-testing method for the initial detection of rebars in large-sized structures (e.g., with the use of a magneto-optical MO sensor) [48].

### 1.2. Knowledge Acquisition from the Experiment

**Figure 2:**
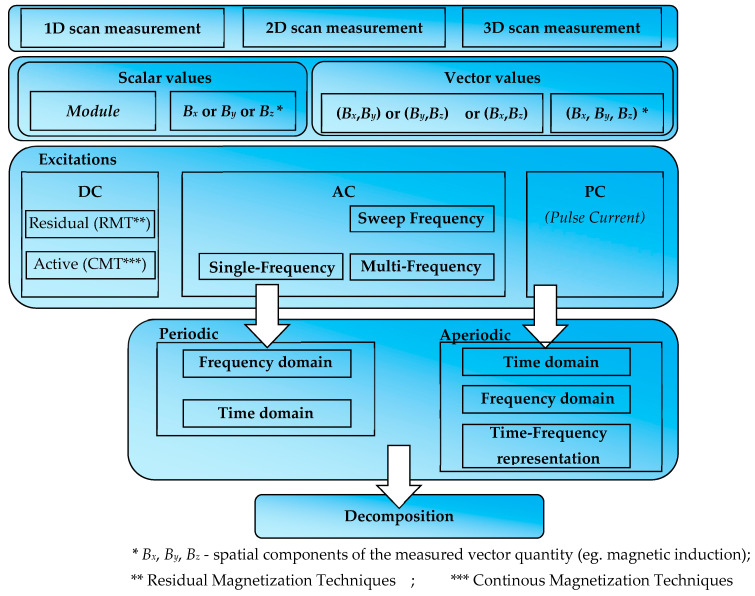
Methods used to obtain more complete information from the experiment.

Signals acquired from any inspection method require interpretation allowing identification of the tested object. Attribute extraction is crucial in the identification process. This task is even more significant in cases where the tested structure is complex, or several parameters are identified at once. There are many methods to achieve more complete information from the experiment results. Basic methods are presented in Figure 2.

The easiest way to achieve complete information from the test (Figure 2) is to increase the area and number of measurements. In this approach, two variants are possible. A single sensor can be moved over the tested object in one, two, or three dimensions (an excellent example of the scanning method is the Total Focusing Method), or many sensors can be used simultaneously in different places to obtain the spatial distribution of the results (multimodal/multi-source). Both methods can also be combined. The technique for aggregating and processing multi-source data is called data fusion. In the case of a single measurement obtained from one position of the single sensor, results may be ambiguous. A series of measurements for different positions create opportunities to identify the object entirely and undoubtedly. The important aspect is choosing the suitable sensor for the measured phenomenon. In the case of vector quantities, more than one spatial component should be analyzed. It allows for a better interpretation of the studied phenomenon.

Another way to increase the amount of information obtained from the measurement is to change the form of excitation. In the case of periodic excitation, a multifrequency (MF) or sweep-frequency (SF) method can be used instead of single-frequency testing. These techniques, e.g., in the eddy current method, allow to utilize the relationship between the frequency and penetration depth. Additionally, the multifrequency approach quickly provides information about resonance frequencies. MF and SF techniques are explored in mechanical (modal analysis [3]) and electromagnetic methods [33,38,39]. Pulse signals can be analyzed in the time and frequency domains (by appropriate transformation, e.g., FFT) or changed into time-frequency representation by wavelet analysis. Multifrequency signals are usually analyzed in the frequency domain, simplifying the analysis [39,44].

Decomposition is an additional option to receive more information from the waveform. For example, the single-frequency signal can be decomposed into amplitude, frequency, phase shift, and offset.

## 2. Materials and Methods

### 2.1. Measuring System for Magnetic Inspection

**Figure 3:**
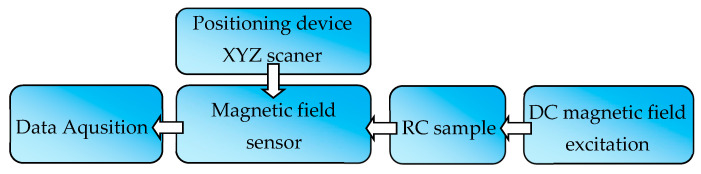
Block scheme of the measuring system.

The measuring system consisted of four subsystems: excitation subsystem, positioning subsystem, magnetic field transducer, and data acquisition subsystem. The block diagram of the system is shown in Figure 3.

**Figure 4:**
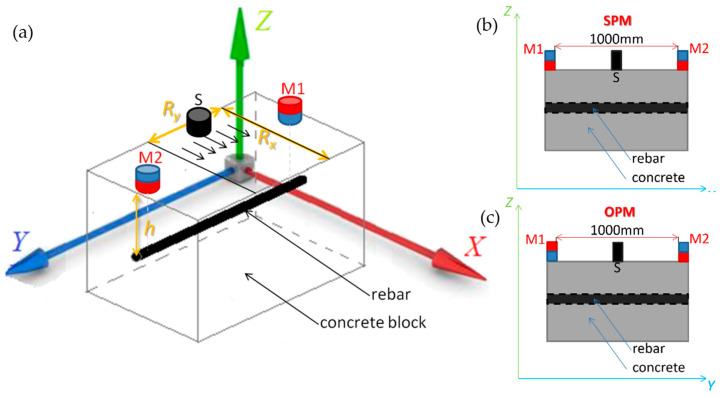
Schematic view of the sample with depicted measurement area; where: *h*—concrete cover thickness, *R_x_* and *R_y_*—the parameters determining the size of the measurement area, M1 and M2—magnets, S—sensor, (**a**) 3D view, (**b**) 2D view—SPM, (**c**) 2D view—OPM.

The excitation system (Figure 4a) consists of two neodymium magnets, M1 and M2, placed on the sample’s surface directly above the rebar (in distance *h*). The magnets can be arranged in two different ways. In the first configuration (OPM—opposite poles magnetization), the magnets have opposite poles facing the sample (Figure 4c). In the other configuration (SPM—same pole magnetization), the magnets were orientated to the sample with the same poles (Figure 4b). The distance between M1 and M2 was determined experimentally and equaled 1000 mm. The selection criteria were, on the one hand, the possibility of magnetizing the rebar in the measurement area and, on the other hand, a small direct influence of the magnets on the sensor. The magnets are not moved during the measurements. In the experiments, the samples are scanned with an AMR sensor (S) moving on the concrete’s surface in two orthogonal directions, *x* and *y* (the size of the scanning area is determined by the parameter *R_x_* and *R_y_*—Figure 4a). The used AMR element (HMC5883L, Honeywell, NJ, USA) is crucial to the system. The sensor enables the detection of magnetic field induction spatial components (*B_x_*, *B_y_*, *B_z_*) and has high sensitivity and field resolution. However, it can be permanently affected by strong magnetic fields.

### 2.2. ACO (Amplitude-Correlation-Offset) Decomposition

#### 2.2.1. Motivation

The magnetic tests can be used similarly to the eddy current (EC) method. In opposition to EC transducers, magnetic sensors can perform area testing. Moreover, the magnetic method is very cheap in implementation (straightforward excitation system) and universal in application. Analyzing particular spatial magnetic components may improve the method’s effectiveness. On the other hand, the highest disadvantage of DC magnetic tests (low spatial resolution) is not that important in the evaluation of relatively huge inhomogeneities (such as rebars in concrete structures) [3,48]. All of these factors make the magnetic method very promising for the task. The biggest challenge in evaluating the RC structure process is the identification of three output parameters based on three input waveforms.

The classic identification process requires a database consisting of a large amount of training data. Usually, the tested waveform is matched to the most similar class from the learning database. In the case of magnetic tests of reinforced concrete structures (RC), obtaining the representative database can be difficult. Rebars are standardized only for mechanical properties (not electromagnetic ones). Therefore, rebars from different steelworks may give different results in magnetic tests. Various admixtures added to the concrete can also influence the results. For these reasons, it may be difficult or even impossible to collect a large number of samples relevant to the case under examination. 

The proposed way to solve the problem of a limited representation in a database is to use ACO (Amplitude-Correlation-Offset) decomposition. In this method, a reference measurement is utilized. Such a measurement can be made on a sample specially made for this purpose or in a place where it has been verified that the object parameters and the materials are appropriate. The measurements from examining the reference and tested samples are compared. Based on the deviations of the results, it is possible to determine how much a given measurement differs from the reference. The ACO decomposition is developed for tasks where technical requirements are known, and the goal is to check how far a given object does not meet the requirements. Moreover, the ACO method enables accurate reflection of the waveform shape and limitation of the attribute number of three or fewer (avoiding the curse of dimensionality). Therefore, the method can be used to identify the parameters of the tested sample with high precision. 

#### 2.2.2. Description of ACO Decomposition

As was mentioned, the identification based on the ACO parameters could be made by matching the tested signal with the most similar class in the database (classic identification with cross-validation) or by comparing the measurement received from the tested object with the measurement obtained from the reference sample, where parameters are known (method utilized in this paper), and testing the deviation (the difference between the ACO parameters obtained for the reference and the tested sample). The algorithm of ACO decomposition consists of two steps:

1. The cross-correlation between the windowed reference and tested signals is calculated in the first step. The results are utilized to determine the window’s lengths and position. The window with the reference signal is moved along the window with the tested signal (by one sample each time), and the cross-correlation is calculated for each setting. The setting when the absolute value of the cross-correlation achieves maximum is taken for further calculations. This step is used to find the rebar position precisely.
2. The second step is devoted to determining the ACO attribute values using the later-mentioned algorithms.

**Offset (*O*)**

The offset (*O*) is a relative quantity calculated by dividing the offset of the test signal (*O*_T_) and the reference signal (*O*_R_). The reference signal, ***R***, and the tested signal, ***T***, are vectors. The offset *O* is calculated using Equation (1).

(1) $$O=\left|\frac{O_{R}-O_{T}}{O_{R}}\right|$$

where:

- absolute offsets of the test signal: *O*_T_ = min(abs(min(***T***)), abs(max(***T***))).
- absolute offsets of the reference signal: *O*_R_ = min(abs(min(***R***)), abs(max(***R***))).

**Amplitude (*A*)**

The relative amplitude *A* is calculated using Equation (2).

(2) $$A=\left|\frac{A_{R}-A_{T}}{A_{R}}\right|$$

where:

- absolute amplitude of the test signal:

*A*_T_ = max(abs(min(***T***)), abs(max(***T***))) − *O*_T_,

- absolute amplitude of the reference signal:

*A*_R_ = max(abs(min(***R***)), abs(max(***R***))) − *O*_R_.

**Correlation (*C*)**

The standard, normalized cross-correlation Formula (3) was used to calculate the correlation parameter *C*. This parameter’s specificity allows for determining a position where the tested signal is similar to the reference signal (in this case, it is synonymous with the position of the rebar). Moreover, correlation can be used to compare the shapes of two waveforms, neglecting their amplitudes. Unfortunately, the signal offset impacts the correlation. Therefore, the offset must be eliminated from all signals before the calculations, giving:

- the corrected tested signal ***TC*** = ***T*** + α*O*_T_,
- the corrected reference signal ***RC*** = ***R*** + α*O*_R_,

where:

(1) α = 1, if all elements of the vector ***T*** are negative OR (elements of the vector have different signs AND abs(min(***T***)) < abs(max(***T***));
(2) α = −1, if all elements of the vector ***T*** are positive OR (elements of the vector have different signs AND abs(min(***T***)) ≥ abs(max(***T***)).

The proposed corrected parameters are independent, increasing the identification effectiveness. 

The standard, normalized cross-correlation is calculated using Equation (3).

(3) $${Corr\_norm}_{RC,TC}=\frac{\sum_{n=0}^{N-1}RC\left[n\right]\cdot TC\left[n\right]}{\sqrt{\sum_{n=0}^{N-1}{RC}^{2}\left[n\right]\cdot \sum_{n=0}^{N-1}{TC}^{2}\left[n\right]}}$$

### 2.3. ACO Decomposition in Comparison with Other Methods of Extracting Attributes

**Figure 5:**
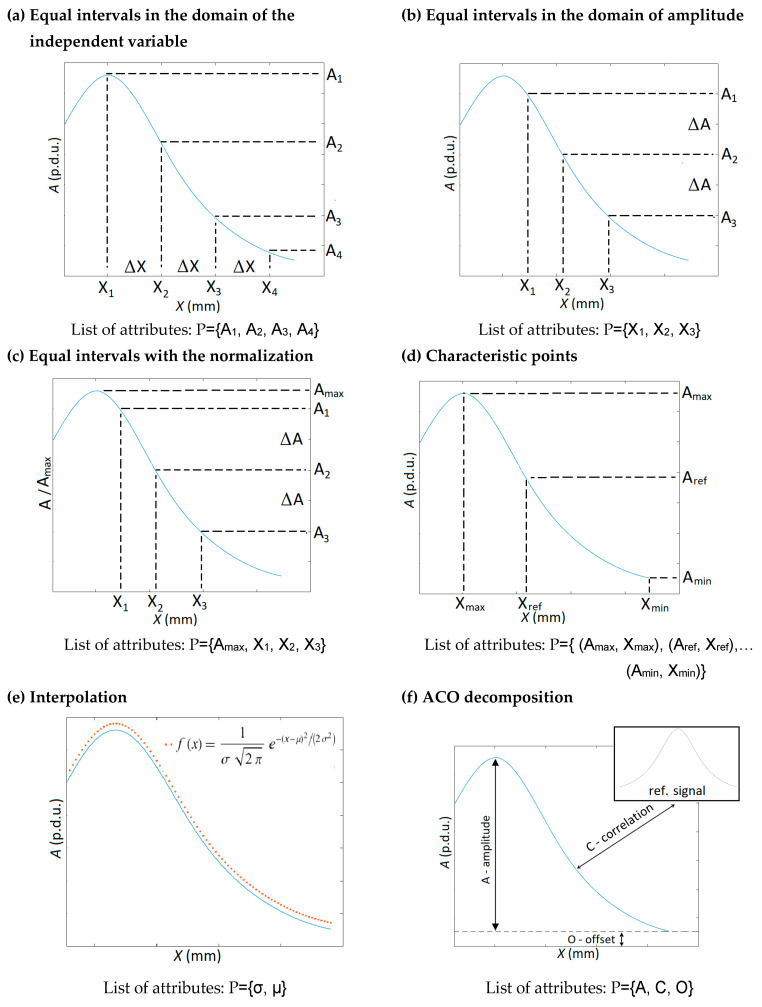
Basic methods to extract attributes from the signal/waveform.

There are many ways to extract attributes from signals. Most of the most popular are variations of the primary methods of extraction presented in Figure 5.

Extraction of attributes from the signal is a complex task. Usually, choosing or finding a compromise between the exact representation of the signal and the minimization of the number of attributes is necessary. Increasing the number of attributes makes knowledge base records increasingly sparse and results less and less reliable. Maintaining the credibility of the results requires that the size of the knowledge base grows exponentially with the number of attributes. This phenomenon is called the curse of dimensionality. 

Such approaches to attribute extraction, such as “equal intervals in the domain of the independent variable” (Figure 5a) or “equal intervals in the domain of amplitude” (Figure 5b), can cause mentioned the curse of dimensionality. To solve this problem, the number of attributes must be initially large and next significantly reduced. Typically, attributes strongly correlated are combined into one or eliminated. At the same time, irrelevant attributes are removed. Then, the significance of the attributes and their correlation matrix are redefined. The process is iteratively repeated until extensive records with many attributes are reduced to records with an optimal number of attributes (usually less than five). There are many methods to eliminate and merge attributes. The basic tools are wrappers and filters [57]. The process of selecting attributes is time-consuming, highly arbitrary, and requires experience to carry it out correctly. PCA and rough set theory are other, slightly more efficient, and less arbitrary methods.

The attributes extraction methods presented in Figure 5a,b pose a few advantages. They do not require prior knowledge of the process under study or database and can be used in any case. The most significant advantage of the “equal intervals in the domain of amplitude” method over the “equal intervals in the domain of an independent variable” is that the number of attributes extracted from the part of the waveform is proportional to the derivative of this segment. However, the result may be inconclusive (one amplitude value may be assigned to more than one *x*).

A significant improvement in identification accuracy can be obtained by making the waveform’s amplitude independent of its shape (“equal intervals with a normalization”—Figure 5c). This procedure can be used for both methods of “equal intervals.” It may be done by determining the waveform’s amplitude and saving it as a separate attribute. Then, the signal is normalized, and shape attributes are extracted. Usually, the attributes selection process is more accessible in this case than in the two previous methods.

The “characteristic points” method utilizes an entirely different approach (Figure 5d). In this case, only distinctive points, not the whole waveform, are reflected in attributes. The number of attributes is small and further processing is straightforward or unnecessary. However, a lot of information about the waveform’s shape is lost. Therefore, the method works well only in standard, foreseen cases. Otherwise, it may give the wrong result.

Additionally, prior knowledge about the data is necessary to properly select attributes (e.g., maximum, minimum, pulse width, distance between extremes, etc.). The method gives excellent identification results in typical cases. The examples of the method application are presented in [33,39].

The “interpolation” method can be considered an improvement and development of the “characteristic points” approach (Figure 5e). Moreover, in this case, only a few attributes are received as an output. This time the attributes are used not to represent characteristic points but the characteristic function of interpolation (entire waveform). The method can be effectively used mainly when the theoretical description of the process (the function) is known. Using interpolation functions, e.g., gauss or polynomial, without knowledge of the theoretical description usually results in low-quality effects, e.g., the number of attributes is large, and/or the shape of the waveform is not well-reflected. The method has many advantages. It allows for generating additional cases (records interpolation), eliminating disturbances/noise, and determining characteristic points.

The already described ACO decomposition (Figure 5f) was created by the evolution of other previously described methods of attribute extraction. Analogically as in the case of the “equal intervals with the normalization” method (Figure 5c), the ACO decomposition separates the amplitude (A) and the offset (O) from the shape attribute (all attributes are independent). In this way, high accuracy of the identification is obtained. The decomposition also refers to the “interpolation” method (Figure 5e). The shape of the waveform is compared to a specific pattern (interpolation—a mathematical function, ACO—a reference measurement) and described by only one parameter, the correlation (C). Therefore, ACO decomposition provides almost all the advantages of the interpolation method while eliminating its most significant disadvantage (no prior knowledge of the waveform shape is required).

### 2.4. Description of the Experiments

In this research, measurements of reinforced concrete structures were carried out in order to identify the object’s parameters. The process may be considered an inverse and an iterative problem involving four (typical for NDT investigations) steps:

1. Designing the hardware (and usually software) layer of the system;
2. Measurements and data processing;
3. Attribute extraction from the obtained data;
4. Identifying the structure parameters.

At each stage of the experiments, the obtained results are controlled, and necessary adjustments are made (if needed). When a problem is found, it is necessary to make corrections in this and often in all subsequent points. Such iterations usually need to be repeated many times.

The first encountered problem was selecting the proper setup of the magnetizing elements. The same pole magnetization (SPM) and the opposite pole magnetization (OPM) are considered in this paper. The advantages and disadvantages of both methods are described in previous work. According to [48], more effective than OPM is SPM, and this configuration is used in further experiments.

All three spatial components (*B_x_*, *B_y_*, *B_z_*) are measured in the second step. In the third step, the waveforms achieved for each component are decomposed by the ACO method into amplitude A, correlation C, and offset O. As a consequence, nine attributes are obtained from each measurement: Ax, Cx, Ox, Ay, Cy, Oy, Az, Cz, and Oz. The significant advantage of ACO decomposition is that the attributes extracted from each waveform can be presented in a clear and easy-to-interpret way using a single 3D graph or three 2D graphs.

The graphs allow a reduction in the number of attributes to the necessary minimum, which is essential for avoiding the curse of dimensionality (Section 2.3).

The last step is identification. Three parameters are estimated: concrete cover thickness *h*, rebar diameter *D*, and rebar class. Typical concrete cover thickness *h* for RC structures ranges from 20 to 50 mm. Therefore, tests are conducted in the range from 20 to 70 mm, with the step of 10 mm (six instances: *h*_20_, *h*_30_, *h*_40_, *h*_50_, *h*_60_, *h*_70_). In reinforced concrete structures, reinforcing bars with 10 and 12 mm diameters are often used. The same rebars (*D*_10_ and *D*_12_) are used during the experiments. The last parameter is the rebar class. For the experiments, the three most popular classes of rebars are selected. According to the national standard PN-B-03264:2002 [58], these rebars are marked as AI (highest flexibility and lowest hardness of the alloy), AIII (low flexibility and high hardness), and AIIIN (lowest flexibility and highest hardness). To summarize, four different rebars are used in the experiments: *D*_10_-AI, *D*_10_-AIII, *D*_12_-AIII, and *D*_12_-AIIIN. The popularity of the materials used in the experiments makes the tests relevant to civil engineering reality. The list of the samples used in the experiments is provided in Table 1.

**Figure 6:**
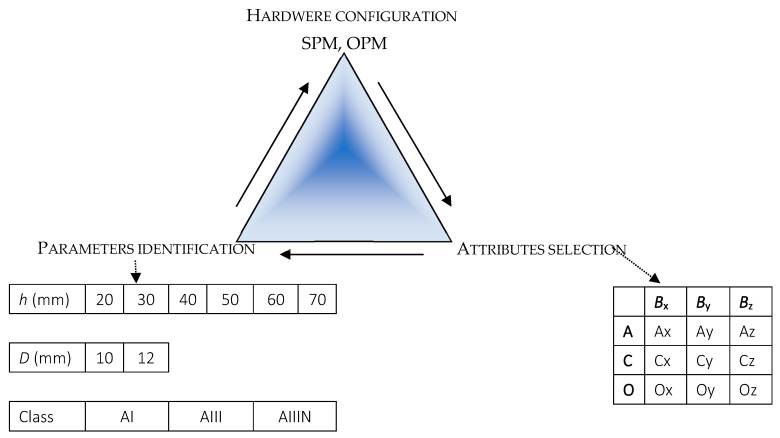
Different stages of the identification process.

As mentioned earlier, the process of identification is usually solved iteratively. The idea is presented in Figure 6. A large number of parameters to be identified and many possibilities for setting input parameters (hardware, software, attribute extraction, etc.) make the identification task complex and challenging to solve. Similar issues have been studied before [32,41,42,43]. In these works, relatively advanced methods have been used, and final results do not always yield satisfactory effects. The ACO decomposition was used to simplify the identification process and improve effectiveness.

Reinforcing bars with a diameter from 10 to 12 mm are usually connected (reinforced grid) into eyes with sides 150 or 200 mm (150 × 150 or 200 × 200 mm). In order to avoid the influence of adjacent rebars on the results, the measuring range was limited. Measurements in the *x*-axis were made in steps of 1 mm in the range of 0 to 96 mm (*R_x_* = 96 mm). Due to the symmetry of the received signals (left and right side of the rebar), the total range measurements were made only in one direction (rebar positioned in *x* = 26 mm). The sensor was also moved in the *y*-axis (*R_y_* = 50 mm; six measurement points spaced every 10 mm). As a result of this movement, six measurements were obtained, which were used in the identification process. The research presented in this work is intended to lead to the construction of the target system. The system will use a multisensor transducer with six AMR elements. The whole system is shown in Figure 4.

## 3. Results

### 3.1. Evaluation of the Concrete Cover Thickness h

**Figure 7:**
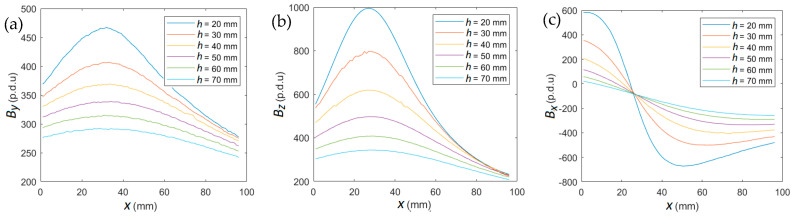
The measurements of spatial components of magnetic induction for sample P2 and magnetization SPM; (**a**) *B_x_* vs. transducer position *x*, (**b**) *B_y_* vs. transducer position *x*, (**c**) *B_z_* vs. transducer position *x*.

In the identification process, spatial components of magnetic induction (*B_x_*, *B_y_*, *B_z_*) were used as a function of the transducer position *x*. The examples of measured waveforms are shown in Figure 7. The presented results refer to sample P2 (rebar: *D*_10_-AIII).

**Figure 8:**
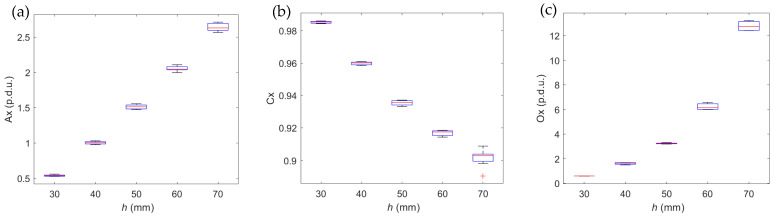
Repeatability of measurements vs. cover thickness—sample P2; magnetization SPM; in this boxplots the central mark indicates the median, the bottom and top edges of the box indicate the 25th and 75th percentiles, respectively. The whiskers extend to the most extreme data points. not considered outliers, and ‘+’ marker symbolize outliers. (**a**) Ax vs. *h*, (**b**) Cx vs. *h*, (**c**) Ox vs. *h*.

**Figure 9:**
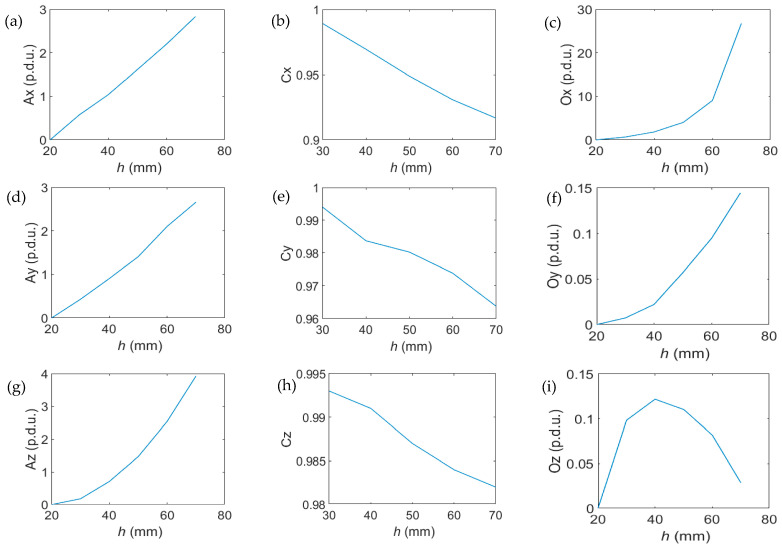
The values of all three parameters (A, C, O) for the three spatial components of magnetic induction (*B_x_*, *B_y_*, *B_z_*) vs. concrete cover thickness *h*; (**a**) Ax vs. *h*, (**b**) Cx vs. *h*, (**c**) Ox vs. *h*, (**d**) Ay vs. *h*, (**e**) Cy vs. *h*, (**f**) Oy vs. *h*, (**g**) Az vs. *h*, (**h**) Cz vs. *h*, (**i**) Oz vs. *h*.

Many factors may affect the results received with the magnetic method. However, the method’s repeatability is high in an environment without external disturbances, as shown in Figure 8. The ACO decomposition is a comparative method in which the waveform under study is related to the reference. In the results presented in Figure 8 and Figure 9, the measurement made for the same sample and concrete cover *h* = 20 mm was used as reference data.

The values of all three parameters (A, C, O) calculated for the three spatial components of magnetic induction (*B_x_*, *B_y_*, *B_z_*) in the function of *h* are shown in Figure 8.

The attribute values as a function of cover thickness *h* change monotonically. Except for correlation C, all attributes can be approximated by exponential functions with high accuracy (slight deviation).

**Figure 10:**
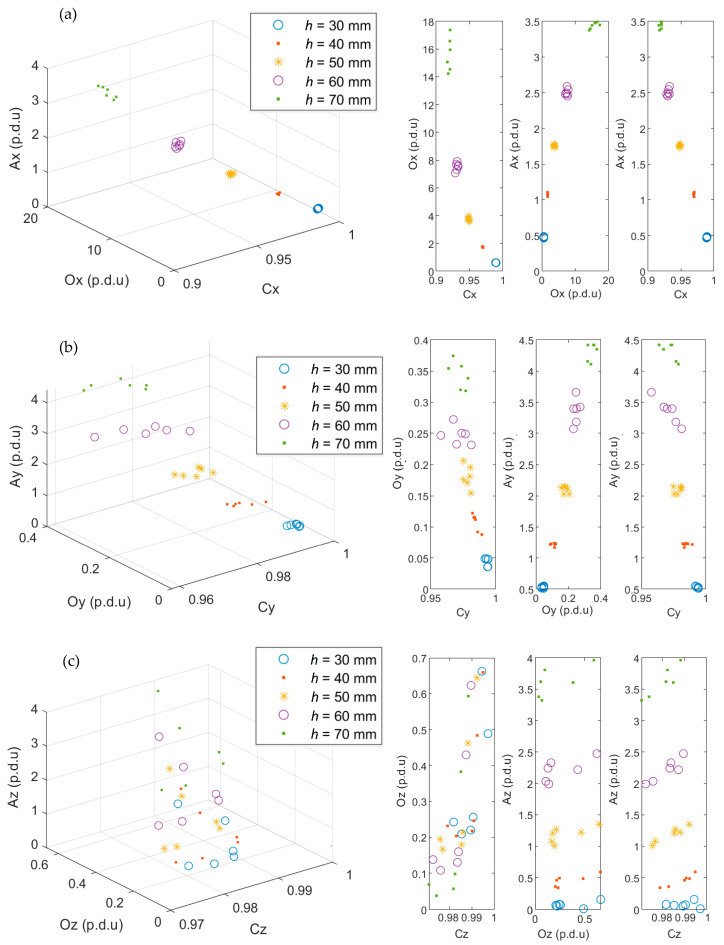
Plots of the parameters (A, C, O) calculated for the three spatial components of the magnetic induction; (**a**) ACO parameters for *B_x_*, (**b**) ACO parameters for *B_y_*, (**c**) ACO parameters for *B_z_*.

The attribute values calculated for all sensor positions on the *y*-axis are presented in Figure 10. The purpose of the graph is to show the separation between clusters corresponding to each concrete cover thickness *h*. The separation between the classes is not always visible in the 3D graphs (Figure 10c). Therefore, the corresponding 2D plots are also presented.

It is visible in the presented results (Figure 10) that the ACO attributes enable identifying concrete cover thickness *h*. The significant separation between the clusters suggests that the identification could be made even with millimeter accuracy. However, such high accuracy is only possible in cases where all other parameters of the RC structure (diameter and class of the reinforcing bar) are known.

### 3.2. Evaluation of the Rebars Diameter D and Class

**Figure 11:**
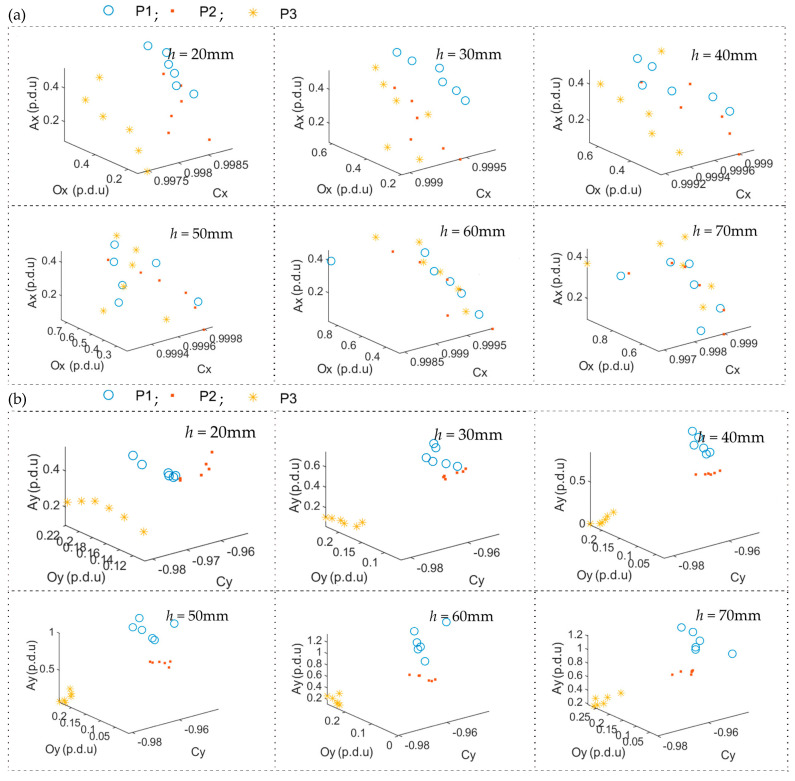

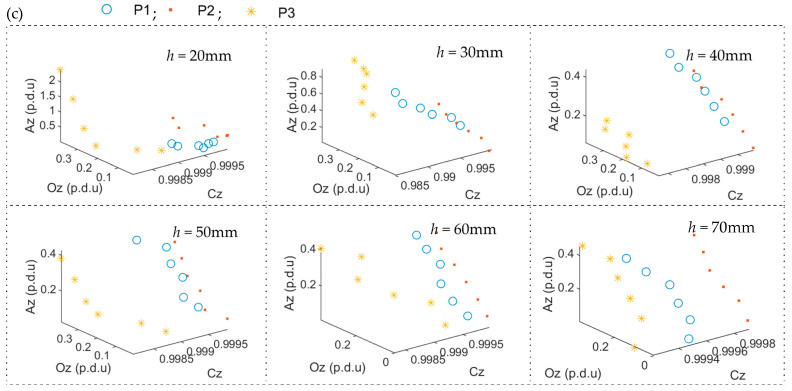
The values of ACO parameters for a specific spatial component of magnetic induction in 3D presentation received for three samples (P1, P2, P3) and constant *h*; P4 used as a reference; (**a**) the component *B_x_*, (**b**) the component *B_y_*, (**c**) the component *B_z_*.

The ability to recognize different reinforcing bars at the same *h* is evaluated by examining the cluster separation. The results of ACO decomposition obtained for different (diameter and class) rebars are presented in Figure 11. In these experiments, sample P4 is used as a reference. As in the case of the measurements shown in Figure 10, the results obtained for six different sensor positions in the *y* direction are plotted.

In the experiment presented in Figure 11, the highest separation is obtained for the spatial component *B_y_* and only slightly worse for *B_z_*. The worst results are observed for the *B_x_* spatial component. It is worth noticing that in the case of *B_x_*, identifying concrete cover thickness *h* above 50 mm is becoming more complex. Similarly, like in the case presented in Figure 10, the most unpredictable and noise-prone attribute is correlation C. High correlation values cause the problem. Since the shapes of all the waveforms are very similar, the correlation is always very high, and even a slight disturbance affects it significantly.

### 3.3. Simultaneous Identification of All Three Parameters (h, D, and Class)

**Figure 12:**
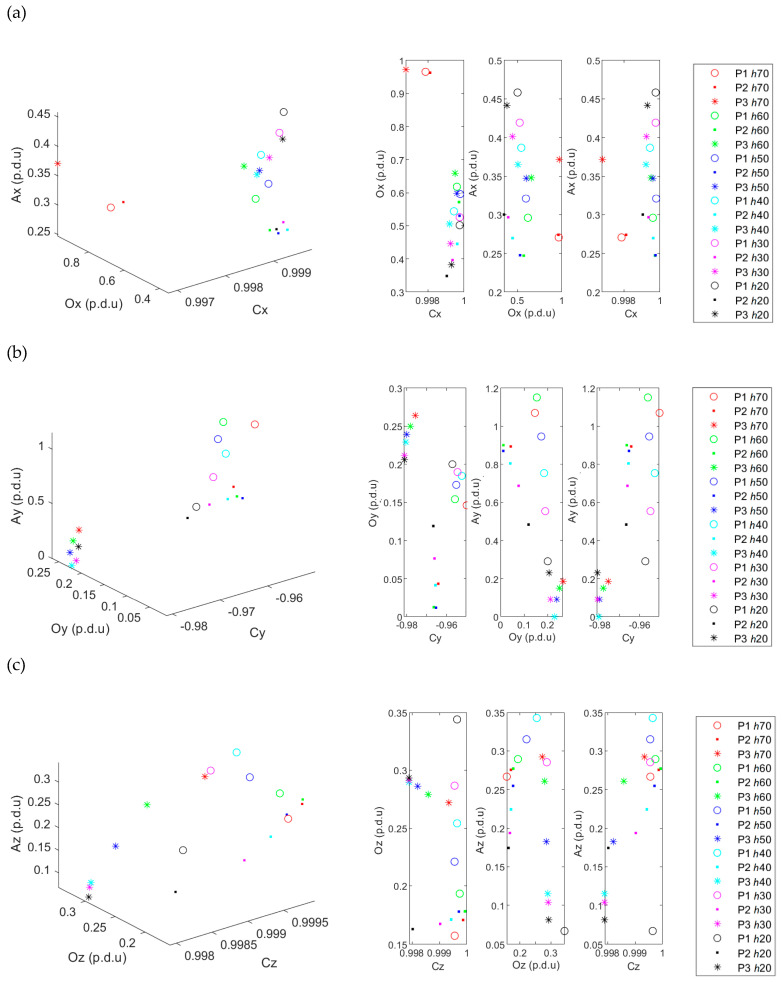
The 3D distribution of ACO parameters calculated for all spatial components of magnetic induction; (**a**) from the component *B_x_*, (**b**) from the component *B_y_*, (**c**) from the component *B_z_*. The results were obtained for the three samples (P1, P2, P3) and different cover thicknesses *h*; sample P4 was used as a reference.

Values of all possible classes (combinations of parameter values) are shown in Figure 12. In order to increase the readability of the graphs in Figure 12, only one measurement for a specific configuration is presented (five different measurements on the *y*-axis presented before having been omitted). It can be concluded from Figure 10 and Figure 11 that using a vector of sensors followed by data fusion would significantly simplify the identification of all three parameters (*h*, *D*, and class). However, even using the measurement from the single sensor position, identification is still possible. In Figure 11, different rebars are depicted by the shapes of point markers. Colors distinguish the variate concrete cover thickness in the same graphs. Therefore, attention should be paid to separating clusters of markers of the same shape (different types of reinforcing bars) and the same color (values of concrete cover thickness *h*).

In Figure 12a, it can be observed that the ACO parameters for *h* = 70 mm deviate from the corresponding clusters. It may indicate a significant deterioration in the method’s efficiency for such a large *h*. The problem mainly concerns the *B_x_* component. The best separation was obtained from the ACO parameters calculated for the *B_y_*.

## 4. Conclusions

The magnetic method presented in the paper can successfully and simultaneously identify all three fundamental parameters of reinforced concrete structures (cover thickness *h* and class and diameter *D* of reinforcing bars). The effectiveness of the method was confirmed to a depth of 70 mm. However, the results indicate that even at greater depths, identification is still possible. The effective measurement range can be enlarged by modifying the magnetizing system (different arrangement of the magnets) and enhanced sensitivity of sensor sensitivity.

The tests presented in the paper showed that in order to identify the parameters of reinforced concrete structures using magnetic methods, it is best to study the spatial component *B_y_*. The *B_z_* component is only slightly less suitable for this purpose. In the case of the *B_x_* component, the results are worse than in the other two cases, but even this component can still be helpful and used in the identification.

In this work, the ACO decomposition made it possible to investigate and analyze a complex issue by only using simple clusterization. A basic visual analysis of the clusters shown in the 3D graphs allows identifying three different parameters (cover thickness *h* and class and diameter *D* of reinforcing bars). It is also possible to examine which ACO attributes and spatial components of induction *B* are best suited for the identification. The promising results were achieved due to a small number of attributes (easy analysis and association rules finding) and independence of attributes (simple associations, no hidden connections) obtained in the ACO decomposition. A similar identification problem was analyzed earlier [31,37], but the method was more complex, and the achieved results were less separated.

In this work, the weakest results were obtained for the attribute of correlation C. It was due to the high waveform similarity and extremely high correlation (about 99%). As a result, even a slight disturbance or noise made a significant difference. This parameter could be better suited to the case where the shapes of the waveforms are not so similar. The amplitude A and offset O can be approximated by exponential functions with high accuracy (slight deviation), making them predictable and thus valuable for identification.

## Figures and Tables

**Table 1:** Parameters of the tested samples.

	P1	P2	P3	P4
Diameter *D* (mm)	10	10	12	12
Class	AI	AIII	AIII	AIIIN
